# Assessment of Nuclear Gem Quantity for Evaluating the Efficacy of Antisense Oligonucleotides in Spinal Muscular Atrophy Cells

**DOI:** 10.3390/mps7010009

**Published:** 2024-01-19

**Authors:** Haya Al-Hilal, Marianna Maretina, Anna Egorova, Andrey Glotov, Anton Kiselev

**Affiliations:** 1 Department of Genomic Medicine Named after V.S. Baranov, D.O. Ott Research Institute of Obstetrics, Gynecology and Reproductology, Mendeleevskaya Line 3, 199034 Saint-Petersburg, Russia; hayahilal.992@gmail.com (H.A.-H.); marianna0204@gmail.com (M.M.); egorova_anna@yahoo.com (A.E.); anglotov@mail.ru (A.G.); 2 Faculty of Biology, Saint-Petersburg State University, Universitetskaya Embankment 7-9, 199034 Saint-Petersburg, Russia

**Keywords:** spinal muscular atrophy, *SMN1* gene, *SMN2* gene, nuclear gems, antisense oligonucleotides, splicing correction

## Abstract

Spinal muscular atrophy is a neuromuscular disorder caused by mutations in both copies of the survival motor neuron gene 1 (*SMN1*), which lead to reduction in the production of the SMN protein. Currently, there are several therapies that have been approved for SMA, with many more undergoing active research. While various biomarkers have been proposed for assessing the effectiveness of SMA treatment, a universally accepted one still has not been identified. This study aimed to describe a fast and reliable method using the number of gems in cell nuclei as a potential tool for assessment of splicing correction of oligonucleotide efficacy in SMA cells. To gain insight into whether the number of gems in cell nuclei varies based on their *SMN* genotype and whether the increase in gem number is associated with therapeutic response, we utilized fibroblast cell cultures obtained from a patient with SMA type II and from a healthy individual. We discovered a remarkable difference in the number of gems found in the nuclei of these cells, specifically when counting gems per 100 nuclei. The SMA fibroblasts treated with antisense oligonucleotide showed beneficial effects in correcting the abnormal splicing of *SMN2* exon 7. It was observed that there was a significant increase in the number of gems in the treated cells compared to the intact SMA cells. The results obtained significantly correlate with an increase of full-length SMN transcript sharing. Based on our findings, we propose using the quantity of gems as a reliable biomarker for SMA drug development.

## 1. Introduction

Spinal muscular atrophy is an autosomal recessive genetic disorder. It is characterized by progressive muscle weakness, which eventually leads to widespread skeletal muscle atrophy due to the consistent degeneration and loss of the α-motor neurons of the spinal cord and lower brain stem [1]. The International SMA Consortium has classified this genetic disorder into four main types, depending on the achieved motor abilities and the age of onset [2], where SMA I is considered the most severe type with onset in the first 6 months of life. SMA type II is an intermediate form, with manifestation within the first 18 months of life; patients with this form are able to sit but none achieve the ability to walk unaided [3]. The milder III and IV types of SMA are characterized by walking ability and normal life expectancy [4]. Although the global carrier frequency is rather high—1 carrier per 40–60 people (average 1 in 50)—SMA is considered as a rare neuromuscular disease, with incidence approximately 1 in 10,000 live births worldwide [5]. SMA is caused by a homozygous deletion of the *SMN1* gene, which leads to decreased expression of survival motor neuron protein SMN [6]. This protein plays a special role in supporting the assembly of spliceosomal U snRNPs and other ribonucleoproteins [7]. This protein is expressed in the human body by two paralogous genes: survival motor neuron genes 1 and 2 (*SMN1* and *SMN2*). *SMN1* gene produces correctly spliced full-length FL-SMN1 transcripts, leading to the production of functional SMN protein almost exclusively. The *SMN2* gene produces mainly alternatively spliced and exon-lacking 7 transcripts (*SMN*Δ7), giving rise to mislocalized, unstable, malfunctioning SMN protein [8]. In all SMA types, the *SMN1* gene product is lacking, and the *SMN2* gene is the only source of SMN protein. Patients with milder forms of SMA usually have a higher number of *SMN2* copies compared to severely affected patients [9]. Thus, SMN protein was also shown to be more detectable in SMA type II patients’ cells compared to SMA type I [10].

Notwithstanding the great breakthrough benefits of the currently existing approaches for SMN-dependent therapies, some major challenges prevail regarding safety issues and targeted organs.

**Figure 1:**
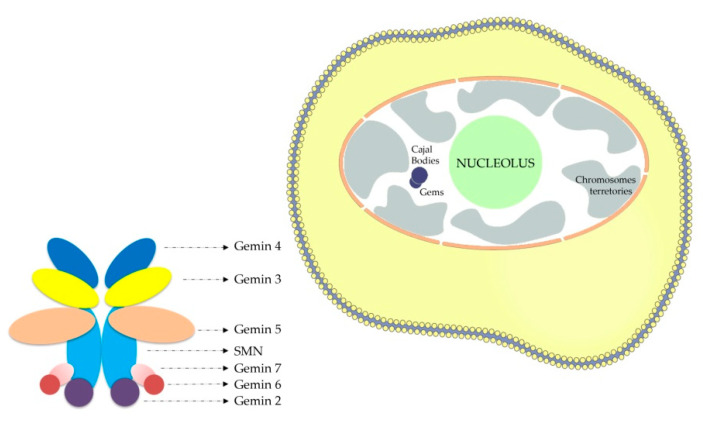
Scheme depicting location of nuclear gems and SMN complex.

Therefore, an urgent need to develop a new therapeutic approach is still of high value as is a powerful biomarker capable of giving accurate diagnostic, prognostic, and predictive information about the response of the treatment and its efficacy and safety. In this study, the number of gems in fibroblast nuclei was tested as a putative biomarker for SMA. A unique characteristic of the SMN protein is its location in speckle nuclear bodies, basically established as “gems”. Gems (or the Gemini of coiled bodies) are nuclear structures that are similar to Cajal bodies (CBs) in size and shape, but they do not contain small ribonuclear proteins, snRNPs [11]. In contrast, “Gems” have SMN protein, which is the affected protein in SMA, and are involved in snRNP maturation. Gems contain high levels of SMN protein and also some SMN-interacting proteins like Gemins (Figure 1). The gem constituents have thus far been restricted to the components of the SMN complex, although Cajal bodies have excess amounts of RNAs and their associated proteins [12]. SMN is a crucial element in the assembly of U-rich small nuclear ribonucleoproteins (snRNPs), which represents the center for splicing. It was reported previously that in the motor neurons of amyotrophic lateral sclerosis patients, the misregulation of snRNP biogenesis was linked to the loss of gems [13]. Other research showed that when the SMN protein was depleted through RNA interference in HeLa PV cells, the gems disappeared completely [13]. This indicates that the SMN protein represents a crucial building block of these Gemini of coiled bodies. Studies performed on different types of cells showed a great difference in the number of gems in the nuclei of cells derived from SMA patients and controls [14,15,16]. Also, an inverse correlation was observed between SMA severity and the number of gems; hence, the increased detection of gems was closely associated with a milder form of the disease [10,14,15].

Gems have been established as a useful means to observe and control the induction of SMN in a diversity of therapeutic molecules, from drugs and to viral vectors. In several studies, it was reported that *SMN2*-inducing histone deacetylase inhibitors (such as benzamide M344, phenylbutyrate, and 4-phenylbutyrate-tethered trichostatin A analogue AR42) and aminoglycosides (such as tobramycin) increased the number of gems in fibroblast cell lines derived from SMA patients [17,18,19,20,21]. Moreover, similar results were observed in iPS-SMA-derived neural cells after treatment with valproic acid and tobramycin [16]. In a study conducted in 2008, it was demonstrated that the fibroblasts transfected with molecule-enhancing trans-splicing of SMN2 transcripts showed a significant increase in gem number [22].

This indicates inverse correlation between the SMN protein levels and SMA severity, and from what was mentioned above, the number of gems as a biomarker for SMA is worth testing. Along these lines, we demonstrated why the number of gems can be considered as a suitable biomarker for SMA.

## 2. Experimental Design

**Figure 2:**
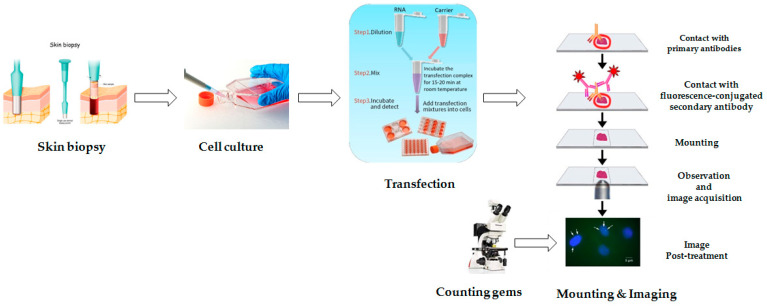
Representative scheme of the protocol for the quantification of the number of nuclear gems.

Here, we present a protocol for the quantification of the number of nuclear gems as a potential biomarker for spinal muscular atrophy. In our protocol, we describe step by step all the procedures as summarized in Figure 2. This protocol will be very useful for researchers who study the molecular mechanisms of SMA pathogenesis and develop new therapeutic approaches. The biomaterials for this study are primary fibroblast cell cultures derived from skin biopsy of a healthy individual as well as a patient of SMA type II [23,24]. The skin biopsy was performed under sterile conditions. All instruments were sterilized by autoclaving before the procedure. The transfection was performed using 3UP8 antisense oligonucleotide to correct the splicing of *SMN2* exon 7 and X-tremeGENE (Roche, Mannheim, Germany) as the transfection reagent, following the producer’s instructions [25]. After the immunocytochemical staining, the cell nuclei were examined under a microscope with a total magnification of ×1000 (Leica, Wetzlar, Germany, DM 2500). The number of gems was counted per 100 nuclei in live mode. Identification of gems was performed by checking fluorescence on the corresponding (green) channel and individual focusing for optimal visibility. This approach is effective because it eliminates the need to individually adjust exposure for the blue and green channels for each photo. Furthermore, the live mode enables us to eliminate any false interpretation of the results by checking “gems” on other channels to eliminate non-specific fluorescence. To reinforce our protocol, we studied the FL-SMN transcripts using semiquantitative RT-PCR and visualized the results using polyacrylamide gel electrophoresis.

### 2.1. Materials

- Single-channel pipettes (0.5–10 µL, 5–50 µL, 20–200 µL, 100–1000 µL) (Lenpipet, Saint-Petersburg, Russia; Cat. No.: 4027482, 40270282, 40270292, 40270302);
- Culture flask 75 cm^2^, for work with adhesive cell cultures (TC treated), lid with filter, sterile (Guangzhou JET Bio-Filtration Co. Ltd., Guangzhou, China Cat. No.: TCF012250);
- Plastic sterile containers, volume 120 mL (Medpolymer, Saint-Petersburg, Russia; Cat. No.: 2620304);
- Micro centrifuge tubes (2.0 mL) (BIOplastics BV, Landgraaf, The Netherlands Cat. No.: B71420);
- Microtubes (1.5 mL) (Axygen, Union City, CA, USA; Cat. No.: MCT-150-C);
- PCR tubes, 0.5 µL (SSI, Lodi, CA, USA, Cat. No.: SSI-3320-00);
- 8-well Permanox chamber slide (Thermo Fisher Scientific, Grand Island, NY, USA; Cat. No.: 177445);
- 24-well plates (TC treated) (Sarstedt AG & Co., Nümbrecht, Germany; Cat. No.: 83.3922);
- Hemocytometer counting chamber;
- 200 μL beveled pipette tips (SSI, Lodi, CA, USA; graduated, yellow, SKU: 4220-06);
- Pipette micro tips (0.1–10 μL) (Jet Biofil, Guangzhou, China; Sterile, Cat. no: PPT151010);
- Filtered pipette tips, sterile (1000 μL) (SSI, Lodi, CA, USA; SKU: 4337NSF);
- Pipette tips (1000 μL) (Jiangsu Huida medical instruments Co., Ltd., Yancheng city, China; Cat. no: HP2036-1);
- Cryotubes, sterile (Deltalab S.L., Barcelona, Spain; Cat. No.: 409106.1);
- Nutrient medium DMEM liquid, with stable glutamine (1 L) (alanyl-glutamine) glucose 1 g/L, sterile (Bilot, Russia; Cat. No.: 1.3.6.3.);
- Fetal bovine serum, Gibco (100 mL) (Thermo Fisher Scientific, Waltham, MA, USA; Cat. No.: 10270-098);
- Penicillin-Streptomycin, for cell culture, dry powder, 50,000 U/vial penicillin G and 50 mg/vial streptomycin, sterile (BIOFIL, Saint-Petersburg, Russia; Cat. No.: 1.3.18.);
- Gibco^®^ Trypsin-EDTA (0.25%), phenol red (Thermo Fisher Scientific, Waltham, MA, USA; Cat. No.: 25200056);
- 3UP8 RNA oligonucleotide 5′-GCUGGCAG-3′ with phosphorothioate and 2′-O-methyl modifications [25] (Syntol JSC, Moscow, Russia);
- X-tremeGENE™ siRNA Transfection Reagent (Roche, Mannheim, Germany; SKU: 4476093001);
- Triton X-100 (Helicon, Moscow, Russia; vendor code: SB-G1204-100ML);
- Bovine serum albumin, BSA, 500g (Sigma-Aldrich, Steinheim, Germany; SKU: A3059-500G);
- SMN antibody (2B1) NB100-1936 Unit Size: 0.1 mL (Novus Biologicals, Littleton, CO, USA; Cat. No.: NB100-1936);
- Donkey Anti-Mouse IgG NorthernLights™ NL493-conjugated Antibody (BioTech R&D systems, Minneapolis, MN, USA; Cat. No.: NL009);
- VECTASHIELD^®^ Antifade Mounting Medium with DAPI (H-1200-10) (Vector laboratories, Burlingame, CA, USA; Cat. No.: H-12000);
- Acrylamide 2K Standard grade, extra-pure, 500 g (PanReac AppliChem, Darmstadt, Germany; product code: A1089).;
- Boric acid, chemical grade, 500 g (Helicon, Moscow, Russia; vendor code: H-0202-0.5);
- EDTA, disodium salt, dihydrate 500 g (Helicon, Moscow, Russia; vendor code: H-E5134-0.5);
- Ethidium bromide (Helicon, Moscow, Russia; vendor code: SRL-17220-5G);
- N,N,N′,N′-Tetramethylethylenediamine TEMED (Helicon, Moscow, Russia; vendor code: SB-GC203001);
- Ammonium persulfate (APS) (Merck; Darmstadt, Germany; Cat. No.: h-248614-0.1);
- 1X 0.25% trypsin solution (BioloT, Saint-Petersburg, Russia; Cat. No.: 1.2.2.5.);
- 0.3% Versen’s solution (BioloT, Saint-Petersburg, Russia; Cat. No.: 1.2.3.2.);
- DPBS without Ca^2+^ and Mg^2+^ (Sigma-Aldrich, Steinheim, Germany; Cat. No.: 59331C-1000ML);
- 96° ethyl alcohol P.O.A. (Merck, Darmstadt, Germany; Cat. No.: 8.18760.1000);
- TRIzol reagent (Invitrogen, Carlsbad, CA, USA; Cat. No.: 15596026);
- Chloroform (Vekton, Saint-Petersburg, Russia);
- Isopropanol (Vekton, Saint-Petersburg, Russia);
- Dimethylsulfoxide (DMSO) (VWR (Amresco), Montreal, QC, Canada; Cat. No.: Am-0231-0.1);
- M-MLV reverse transcriptase (Sileks, Moscow, Russia; Cat. No.: E1211);
- Random hexa primer, 15 o.u./mL (Sileks, Moscow, Russia; Cat. No.: D0310);
- Mixture of dNTP, 25 mM each (Syntol, Moscow, Russia; Cat. No.: dNTP-100-010);
- Taq polymerase, buffer without Mg^2+^ and 25 mM MgCl_2_ (Syntol, Moscow, Russia; Cat. No.: E0120).

### 2.2. Equipment

- Biosafety Cabinet Class II (Laminar systems, Miass, Russia; Cat. No.: 1R-D.001-12ada);
- DNA/RNA UV-cleaner box UVT-S (BIOSAN, Riga, Latvia);
- Ultraviolet germicidal irradiator (recirculator) Desar (Himmed, Moscow, Russia; Cat. No.: av345);
- Centrifuge for 15 mL tubes up to 2300 g (ELMI Ltd., Riga, Latvia; Cat. No.: Elmi CM-6M);
- Centrifuge MiniSpin (Eppendorf, Hamburg, Germany; Cat. No.: 00000030762);
- CO_2_ incubator MCO-19AIC (UV) (SANYO Electr.Co., Ltd., Osaka, Japan; Cat. No.: SA-MCO19);
- Incubator +37 °C (Memmert, Schwabach, Germany; Cat. No.: 9537930);
- Two-compartment refrigerator: +4 °C and −20 °C (POZIS, Zelenodolsk, Russia; Cat. No.: 00000031036);
- Refrigerator −80 °C (SANYO Electr.Co., Ltd., Osaka, Japan; Cat. No.: MDF-U32V);
- Inverted microscope MIBR with a digital camera (LOMO, Saint-Petersburg, Russia; Cat. No.: 00000074356);
- Centrifuge mini-vortex microspin (BIOSAN, Riga, Latvia; Cat. No.: 00000026197);
- Refrigerated centrifuge 5417R (Eppendorf, Hamburg, Germany; Cat. No.: 5407000317);
- UV transilluminator (Vilber-Lourmart, Marné La Vallée, France);
- Camera for vertical electrophoresis (Helicon, Moscow, Russia; Cat. No.: VE-20);
- Microscope Leica, DM 2500 (Leica, Wetzlar, Germany).

## 3. Procedure

### 3.1. Fibroblast Transfection


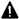
**CRITICAL STEP:** Twenty minutes before handling the material, turn on the UV lamp and Dezar in the Biosafety Cabinet Class II. Ensure that all the procedures are performed under sterile conditions.

1. Twenty-four hours prior to transfection: Culture the healthy fibroblast cells as well as those obtained from the patient with SMA II into an 8-well Permanox chamber slide (for protein analysis) or 24-well plate (for transcripts analysis) in DMEM medium with L-glutamine and 10% fetal bovine serum to reach ~50% confluency per well on the day of transfection (volume 250 μL for each well of 8-well Permanox chamber slide and volume 500 μL for 24-well plate). Incubate the plate with cells in a CO_2_ incubator at 37° C and 5% CO_2_.
2. After 24 h, prepare the transfection complexes of the 3UP8 antisense oligonucleotide with the carrier according to the following: the ratio of X-treme GENE (µL) to RNA (µg) should be 10:1; the molar concentration of 400 nM of the oligonucleotide per well is recommended.
3. Add the complexes of the RNA and the carrier to the cells.
4. Incubate in the CO_2_ incubator at 37° C in 5% CO_2_ for 4 h.
5. After that, change the medium in all wells to full DMEM with 10% FBS and antibiotic (penicillin 100 U/mL, streptomycin 100 μg/mL). Add 250 μL of the full medium in each well of the 8-well Permanox chamber slide and 500 μL in each well of the 24-well plate.
6. Incubate the plates or slides with cells in a CO_2_ incubator at 37 °C and 5% CO_2_ for 48 h.

### 3.2. Immunocytochemical Staining

- Fixation, permeabilization, and blocking:
  1. Remove all the medium from the slide wells, and wash the cells with Dulbecco’s PBS without Ca^2+^ and Mg^2+^ (1x) (about 100 µL volume for each well). After each wash, all the drops should be removed with pipetting. Then, add 50 µL of 4% paraformaldehyde–PBS in each well and incubate for 10 min at room temperature (RT) to allow cells to be fixed into the bottom of the slide.
  2. Remove all the liquid, and wash the cells with PBS. Repeat this procedure one more time.
  3. Add 50 µL of 0.1% Triton X-100 in PBS in each well, and incubate at RT for 5 min for permeabilization.
  4. Remove all the liquid, and wash the cells again with PBS;
  5. Add 50 µL of 1% BSA (Albumin, bovine serum) freshly prepared in PBS, and incubate for 1 h at RT to reduce the background fluorescence.
- Antibody Incubation:
  6. Prepare primary mouse anti-SMN (2B1) antibody dilution (5 µg/mL) in 1% BSA.
  7. Remove all the liquid from the wells, and add 50 µL of primary antibody solution.
  8. Incubate the cells overnight at 4 °C with the primary antibody.
  9. Next day: remove all the liquid from the wells, and wash the cells with PBS three times for 5 min each.
  10. Prepare a dilution of secondary antibody Anti-Mouse IgG NL493-conjugated Donkey antibody (1:200 in PBS).
  11. Add 50 µL of secondary antibody solution in each well.
  12. Incubate for 1 h at RT with secondary antibody in a dark environment.
- Mounting and Imaging:
  13. Remove all the liquid, and wash the cells in PBS three times, each time for 5 min.
  14. Take away the upper part of the chamber to obtain flat slides for microscope analysis.
  15. Mount the cells in Vectashield with DAPI to stain the nuclei (about 10 µL for each well).
  16. Put on the coverslip, and seal with nail polish.

Finally, all the cell nuclei are ready to be examined under microscope with a total magnification of ×1000 (Leica, DM 2500). Search for the nuclei on the blue channel, then check for gems on the green one. Analysis is performed in the live mode to save time and avoid mistakes connected with gem identification. Working with a fine focus while counting gems contributes to a more accurate estimation of their number, because each gem may need individual focusing for a better view. To avoid false interpretation of the results, dots considered as gems may be checked using different filters to be sure they fluoresce on the green channel solely. Dots that fluoresce on different channels should be excluded as debris.

### 3.3. RNA Isolation and cDNA Synthesis


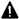
**CRITICAL STEP:** The following protocol is to be performed in a DNA/RNA UV-cleaner box.

1. Remove the medium from the plate, then wash with 200 μL of 1x PBS (without Ca^2+^, Mg^2+^).
2. Add 200 μL trypsin-Versene solution (1:3) to each well to detach the cells.
3. Incubate for 10 min in a CO_2_ incubator at +37 °C.
4. Inactivate the trypsin by adding 300 μL of PBS to each well.
5. Resuspend the cells, then transfer them into 1.5 mL tubes and centrifuge at 2200 r.p.m. for 10 min at +4 °C.
6. Remove the supernatant, add 125 μL of TRIzol reagent to each tube, resuspend, and incubate for 5 min at RT.
7. After that, add 25 μL of chloroform; mix and incubate for 3 min at RT.
8. Centrifuge the resulting mixture at 12,000 r.p.m. for 15 min at +4 °C.
9. Place the upper transparent phase in 1.5 mL tubes, then add 63 μL of isopropanol.
10. Stir the tubes, and place them overnight at −70 °C.
11. Next day: Centrifuge the mixture at 10,000 r.p.m. for 20 min at +4 °C.
12. Remove the supernatant, and wash the residue with 125 μL of cooled 70% ethanol.
13. Centrifuge the tubes at 14,000 r.p.m. for 4 min at +4 °C, and then dry the residue for an hour.
14. Add 20 μL of water treated with diethyl pyrocarbonate (DEPC) to the residue, and dissolve within 40 min by periodically stirring on a vortex.
15. Using a first strand cDNA synthesis kit and random primers, 700 ng of total RNA should be reverse-transcribed following the manufacturer’s protocol instructions.

### 3.4. Semiquantitative RT-PCR

1. Prepare the PCR mix: 1 µL of 10x PCR buffer with MgCl_2_, 0.31 mM of each dNTP, 1 µM of each primer, and 5 U of Taq DNA polymerase.
2. Add 1 µL of cDNA from the total volume of 28 µL to the PCR mix.
3. We recommend using the following primers for full-length and Δ7 SMN transcript amplification: SMN F 5′-GTCCAGATTCTCTTGATGAT-3′, complementary to SMN exon 6 region, and SMN R 5′-CTATAACGCTTCACATTCCA-3′, complementary to SMN exon 8 region [26].
4. The amplification reaction is to be conducted at 94 °C for 4 min, with n cycles of 94 °C for 45 s, 50 °C for 45 s, 72 °C for 45 s, and final synthesis at 72 °C for 8 min.
5. The number of cycles (n) should not exceed 26–28 to stop the reaction on the exponential phase.
6. Amplification of each cDNA sample should be performed at least 2 times.

### 3.5. Polyacrylamide Gel Electrophoresis

To visualize the PCR results, a 6% polyacrylamide gel of the following composition is recommended: TBE 10X—4 mL, Acrylamide solution 30%—9 mL, total volume 40 μL, adding 450 μL of APS and 40 μL of TEMED for gel polymerization).

1. Perform 6% polyacrylamide gel electrophoresis for 80 min at voltage 360 V and current 80 mA.
2. Stain the gel in a solution of ethidium bromide (0.5 μg/mL).
3. The results of polyacrylamide gel electrophoresis are to be recorded on a UV transilluminator.
4. Electrophoresis is processed using densitometric analysis in the software ImageJ v. 1.54d (NIH, Bethesda, MD, USA).
5. The proportion of full-length SMN transcripts as well as the correlation factor are calculated using the program Microsoft^®^ Office Excel^®^ 2007.

### 3.6. Statistical Analysis

Statistical analysis of gem number was performed using the GraphPad Prism 8.0.2 (GraphPad Software Inc., La Jolla, CA, USA). Statistical tests included the Mann–Whitney test. All experiments are represented as median with interquartile range.

## 4. Expected Results

**Figure 3:**
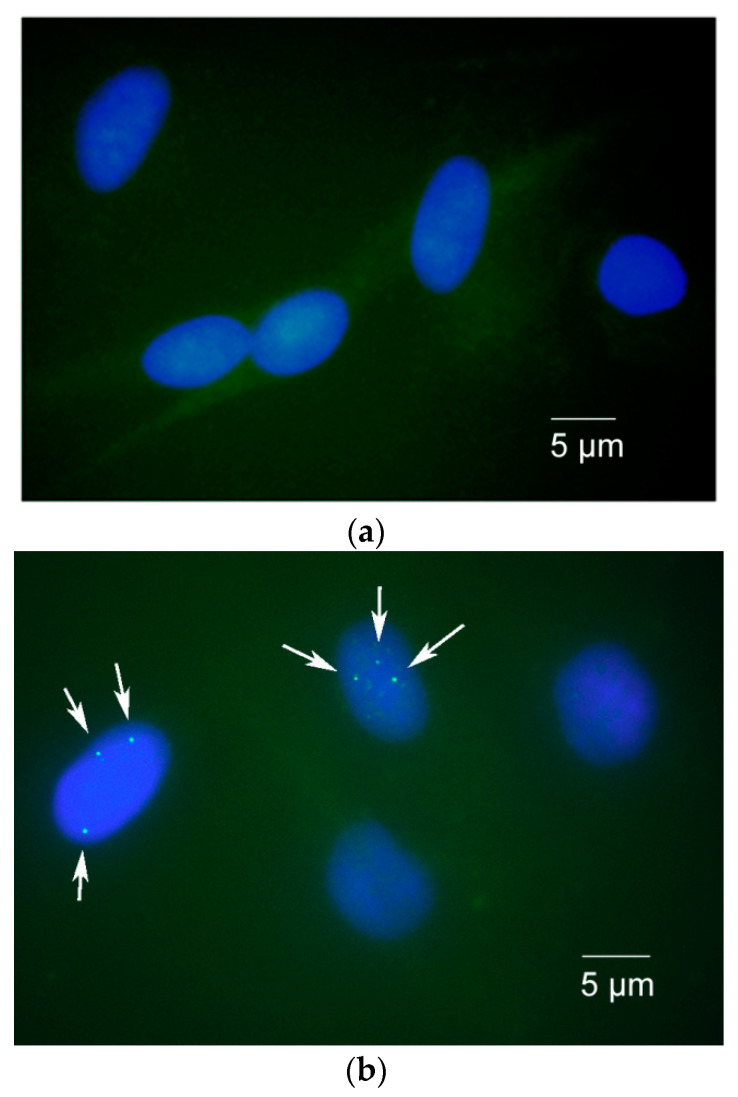

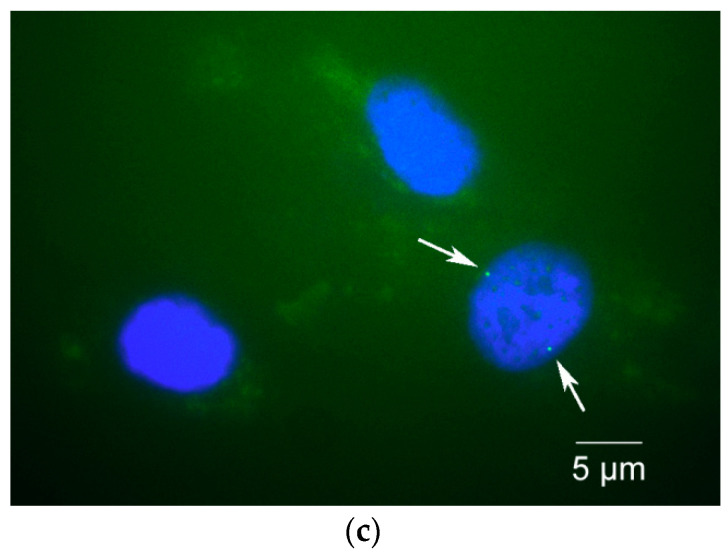
Gems in the nuclei of fibroblast cells of different genotypes: (**a**) nuclei of SMA patient type II cells showing lack of gems; (**b**) nuclei of cells derived from healthy individual showing greater number of gems (arrow); (**c**) nuclei of SMA patient type II cells treated with 3UP8 increased the number of gems related to intact SMA cells (arrow). Scale bar is 5 µm.

The latest therapeutic approaches in SMA have shown encouraging results. Nevertheless, there is still a vital necessity to comprehend and identify the role of biomarkers in the onset of the disease and its development. Quantifying the number of gems in the nuclei of fibroblast cell cultures derived from SMA patients as well as fibroblast cells derived from healthy individuals can serve as a potential biomarker for SMA (Figure 3).

**Figure 4:**
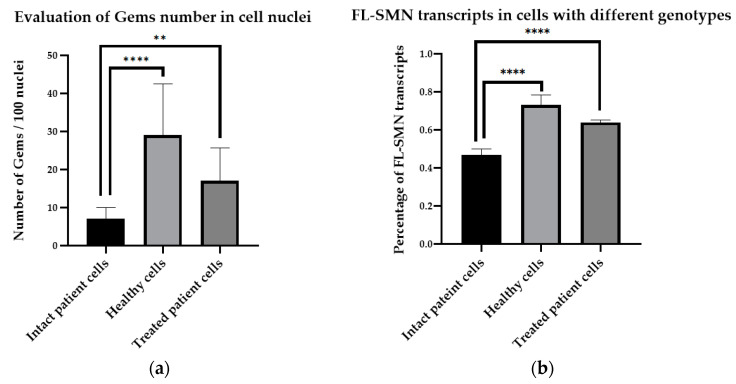
Determination of gem number and FL-SMN transcript percentage: (**a**) differences in the number of gems in the nuclei of cells of different genotypes revealed by Mann–Whitney test (intact SMA II patient cells (n = 24), healthy cells (n = 21), and SMA II patient cells treated with 3UP8 oligonucleotide (n = 8)) per 100 nuclei; (**b**) FL-SMN transcripts in cells of different genotypes (intact SMA II patient cells (n = 22), healthy cells (n = 12), and SMA II patient cells treated with 3UP8 (n = 8)). **—*p* < 0.01, ****—*p* < 0.0001. Medians with interquartile range are given.

**Figure 5:**
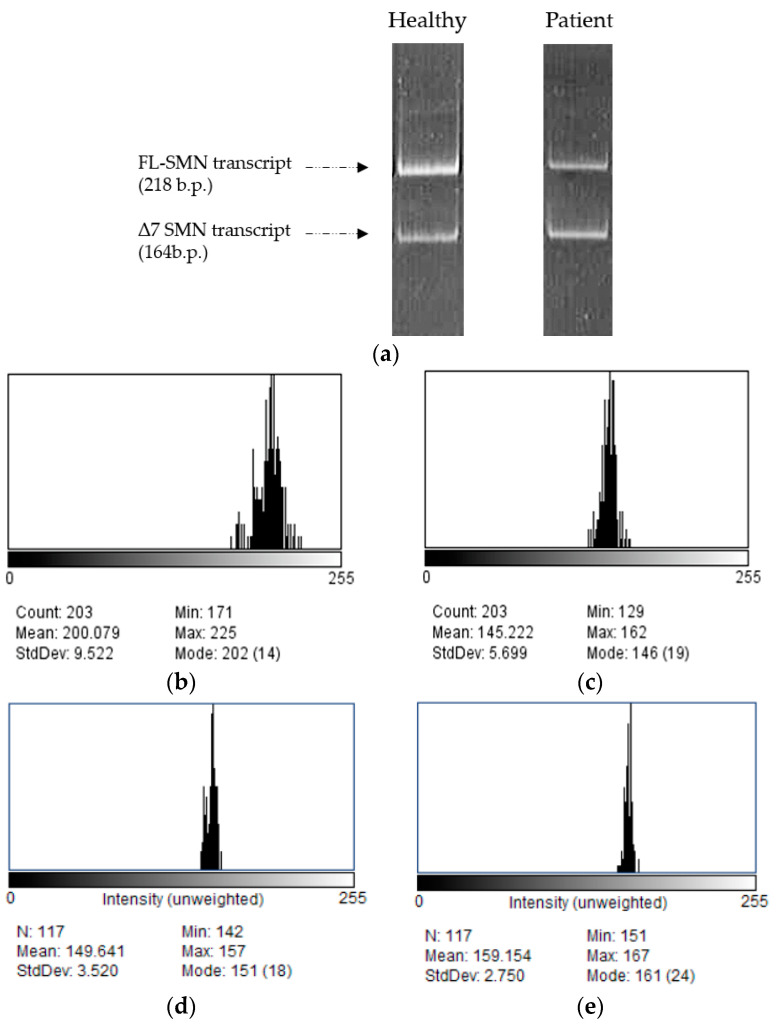
Examples of full-length and exon 7-deleted SMN transcript PCR products of healthy and SMA II patient fibroblasts visualized by gel-electrophoresis (**a**) and analysis of bands from gel-electrophoresis corresponding to full-length and exon 7-deleted SMN transcript PCR products from healthy cells (**b**,**c**) and SMA cells (**d**,**e**) in ImageJ. “Mean” value indicates the intensity of band shining: (**b**,**d**) FL-SMN transcripts; (**c**,**e**) Δ7 SMN transcripts.

It should be noted that there is a significant difference between the number of gems in cells with different genotypes, as the number of gems in healthy cells was significantly higher (29 gems per 100 nuclei, ranging from 15 to 55 per 100 nuclei) than the number detected in the SMA patient’s fibroblast cells (7 gems per 100 nuclei, ranging from 2 to 19 per 100 nuclei) (*p* < 0.0001) according to our findings. To support our protocol, we treated SMA fibroblasts with antisense oligonucleotide 3UP8, previously shown to have therapeutic effects on the level of SMN protein [25]. We discovered a remarkable rise in the number of gems, with up to 17 found in the nuclei of treated patient cells, totaling 100 nuclei, in contrast to the quantity of gems observed in the intact patient cells (*p* = 0.0034) (Figure 4a). The mean value of full-length SMN transcript percentage for each sample was determined by calculating the ratio of the values acquired in ImageJ software for FL-SMN transcripts to the total sum of the values of (FL-SMN + ∆7 SMN) transcripts, based on the fluorescence intensity of the bands relative to the background (Figure 5). The median percentage of FL-SMN transcripts was 0.73 for cells of healthy individuals, 0.64 for cells treated with 3UP8, and 0.47 for cells obtained from the SMA II patient (Figure 4b).

The correlation between the level of full-length SMN transcripts and the number of gems in cell nuclei with different genotypes (intact SMA II patient cells, SMA II patient cells treated with 3UP8, and healthy cells) per 100 nuclei was confirmed to be very strong, with a correlation factor = 0.98 (Figure 4b).

Thus, it can be concluded that the presented protocol describes in detail the assessment of nuclear gem quantity and is suitable for the screening of SMA drug candidates. Previously reported protocols depend on numerous microscopic images, thus slowing gem quantification. In the presented protocol, the identification of gems is performed in live mode, which makes the analysis faster and more precise.

## Data Availability

The data are not publicly available due to restrictions of the subjects’ agreement.

## References

[1] D’Amico A., Mercuri E., Tiziano F.D., Bertini E. (2011). Spinal muscular atrophy. Orphanet J. Rare Dis..

[2] Wirth B., Brichta L., Hahnen E. (2006). Spinal Muscular Atrophy: From Gene to Therapy. Semin. Pediatr. Neurol..

[3] Zerres K., Wirth B., Rudnik-Schöneborn S. (1997). Spinal muscular atrophy—Clinical and genetic correlations. Neuromuscul. Disord..

[4] Russman B.S. (2007). Spinal muscular atrophy: Clinical classification and disease heterogeneity. J. Child Neurol..

[5] Mercuri E., Sumner C.J., Muntoni F., Darras B.T., Finkel R.S. (2022). Spinal muscular atrophy. Nat. Rev. Dis. Prim..

[6] Lefebvre S., Bürglen L., Reboullet S., Clermont O., Burlet P., Viollet L., Benichou B., Cruaud C., Millasseau P., Zeviani M. (1995). Identification and characterization of a spinal muscular atrophy-determining gene. Cell.

[7] Pellizzoni L., Yong J., Dreyfuss G. (2002). Essential role for the SMN complex in the specificity of snRNP assembly. Science.

[8] Monani U.R., Lorson C.L., Parsons D.W., Prior T.W., Androphy E.J., Burghes A.H.M., McPherson J.D. (1999). A single nucleotide difference that alters splicing patterns distinguishes the SMA gene SMN1 from the copy gene SMN2. Hum. Mol. Genet..

[9] Wirth B., Brichta L., Schrank B., Lochmüller H., Blick S., Baasner A., Heller R. (2006). Mildly affected patients with spinal muscular atrophy are partially protected by an increased SMN2 copy number. Hum. Genet..

[10] Patrizi A.L., Tiziano F., Zappata S., Donati M.A., Neri G., Brahe C. (1999). SMN protein analysis in fibroblast, amniocyte and CVS cultures from spinal muscular atrophy patients and its relevance for diagnosis. Eur. J. Hum. Genet..

[11] Liu Q., Dreyfuss G. (1996). A novel nuclear structure containing the survival of motor neurons protein. EMBO J..

[12] Staněk D., Neugebauer K.M. (2006). The Cajal body: A meeting place for spliceosomal snRNPs in the nuclear maze. Chromosoma.

[13] Cacciottolo R., Ciantar J., Lanfranco M., Borg R.M., Vassallo N., Bordonné R., Cauchi R.J. (2019). SMN complex member Gemin3 self-interacts and has a functional relationship with ALS-linked proteins TDP-43, FUS and Sod1. Sci. Rep..

[14] Coovert D.D., Le T.T., McAndrew P.E., Strasswimmer J., Crawford T.O., Mendell J.R., Coulson S.E., Androphy E.J., Prior T.W., Burghes A.H.M. (1997). The survival motor neuron protein in spinal muscular atrophy. Hum. Mol. Genet..

[15] Lefebvre S., Burlet P., Liu Q., Bertrandy S., Clermont O., Munnich A., Dreyfuss G., Melki J. (1997). Correlation between severity and SMN protein level in spinal muscular atrophy. Nat. Genet..

[16] Ebert A.D., Yu J., Rose F.F., Mattis V.B., Lorson C.L., Thomson J.A., Svendsen C.N. (2009). Induced pluripotent stem cells from a spinal muscular atrophy patient. Nature.

[17] Mattis V.B., Rai R., Wang J., Chang C.W.T., Coady T., Lorson C.L. (2006). Novel aminoglycosides increase SMN levels in spinal muscular atrophy fibroblasts. Hum. Genet..

[18] Andreassi C., Angelozzi C., Tiziano F.D., Vitali T., De Vincenzi E., Boninsegna A., Villanova M., Bertini E., Pini A., Neri G. (2004). Phenylbutyrate increases SMN expression in vitro: Relevance for treatment of spinal muscular atrophy. Eur. J. Hum. Genet..

[19] Wolstencroft E.C. (2005). A non-sequence-specific requirement for SMN protein activity: The role of aminoglycosides in inducing elevated SMN protein levels. Hum. Mol. Genet..

[20] Riessland M., Brichta L., Hahnen E., Wirth B. (2006). The benzamide M344, a novel histone deacetylase inhibitor, significantly increases SMN2 RNA/protein levels in spinal muscular atrophy cells. Hum. Genet..

[21] Lumpkin C.J., Harris A.W., Connell A.J., Kirk R.W., Whiting J.A., Saieva L., Pellizzoni L., Burghes A.H.M., Butchbach M.E.R. (2023). Evaluation of the orally bioavailable 4-phenylbutyrate-tethered trichostatin A analogue AR42 in models of spinal muscular atrophy. Sci. Rep..

[22] Coady T.H., Baughan T.D., Shababi M., Passini M.A., Lorson C.L. (2008). Development of a single vector system that enhances Trans-splicing of SMN2 transcripts. PLoS ONE.

[23] Grigor’eva E.V., Valetdinova K.R., Ustyantseva E.I., Shevchenko A.I., Medvedev S.P., Mazurok N.A., Maretina M.A., Kuranova M.L., Kiselev A.V., Baranov V.S. (2016). Neural differentiation of patient-specific induced pluripotent stem cells from patients with a hereditary form of spinal muscular atrophy. Genes Cells.

[24] Nozdracheva A., Pleskach N., Kuranova M. (2020). Features of DNA repair in dermal fibroblasts in patients with breast cancer and persons with medical history of cancer. RAP Conf. Proc..

[25] Singh N.N., Shishimorova M., Cao L.C., Gangwani L., Singh R.N. (2009). A short antisense oligonucleotide masking a unique intronic motif prevents skipping of a critical exon in spinal muscular atrophy. RNA Biol..

[26] Maretina M., Egorova A., Lanko K., Baranov V., Kiselev A. (2022). Evaluation of Mean Percentage of Full-Length SMN Transcripts as a Molecular Biomarker of Spinal Muscular Atrophy. Genes.

